# DNA Binding and Phosphorylation Regulate the Core Structure of the NF-κB p50 Transcription Factor

**DOI:** 10.1007/s13361-018-1984-0

**Published:** 2018-06-05

**Authors:** Matthias Vonderach, Dominic P. Byrne, Perdita E. Barran, Patrick A. Eyers, Claire E. Eyers

**Affiliations:** 1 Centre for Proteome Research, Department of Biochemistry, Institute of Integrative Biology, University of Liverpool, Crown Street, Liverpool, L69 7ZB UK; 2 Department of Biochemistry, Institute of Integrative Biology, University of Liverpool, Crown Street, Liverpool, L69 7ZB UK; 3 Michael Barber Centre for Collaborative Mass Spectrometry, Manchester Institute of Biotechnology, The University of Manchester, 131 Princess Street, Manchester, M1 7DN UK

**Keywords:** Native MS, Ion mobility-mass spectrometry, NF-κB, Collision-induced unfolding, Phosphorylation, DNA binding, Molecular modelling

## Abstract

The NF-κB transcription factors are known to be extensively phosphorylated, with dynamic site-specific modification regulating their ability to dimerize and interact with DNA. p50, the proteolytic product of p105 (NF-κB1), forms homodimers that bind DNA but lack intrinsic transactivation function, functioning as repressors of transcription from κB promoters. Here, we examine the roles of specific phosphorylation events catalysed by either protein kinase A (PKA_c_) or Chk1, in regulating the functions of p50 homodimers. LC-MS/MS analysis of proteolysed p50 following in vitro phosphorylation allows us to define Ser328 and Ser337 as PKA_c_- and Chk1-mediated modifications, and pinpoint an additional four Chk1 phosphosites: Ser65, Thr152, Ser242 and Ser248. Native mass spectrometry (MS) reveals Chk1- and PKA_c_-regulated disruption of p50 homodimer formation through Ser337. Additionally, we characterise the Chk1-mediated phosphosite, Ser242, as a regulator of DNA binding, with a S242D p50 phosphomimetic exhibiting a > 10-fold reduction in DNA binding affinity. Conformational dynamics of phosphomimetic p50 variants, including S242D, are further explored using ion-mobility MS (IM-MS). Finally, comparative theoretical modelling with experimentally observed p50 conformers, in the absence and presence of DNA, reveals that the p50 homodimer undergoes conformational contraction during electrospray ionisation that is stabilised by complex formation with κB DNA.

**Electronic supplementary material:**

The online version of this article (10.1007/s13361-018-1984-0) contains supplementary material, which is available to authorized users.

**Graphical Abstract:**
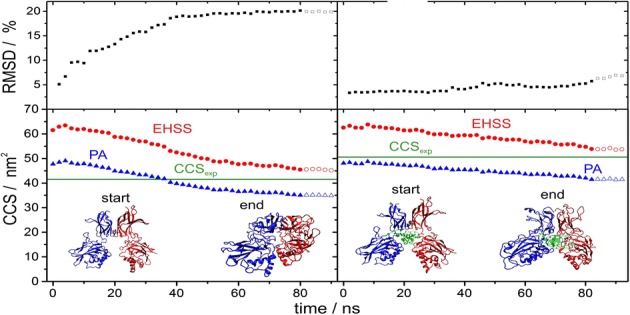
ᅟ

## Introduction

Regulated binding of specific transcription factor complexes to their cognate DNA sequences directly influences the rate at which transcription of individual genes occurs. One such family of ubiquitous transcription factors is NF-kappaB (NF-κB), and regulated activation of this signal transduction pathway is required for transcriptional control of hundreds of genes, under both physiological and pathophysiological conditions. This important family of transcription factors is essential for numerous diverse biological functions, including regulation of inflammation and immune responses, proliferation and apoptosis [1].

Stable interaction of NF-κB (and other) transcription factors with DNA response elements typically requires the formation of either homo- or heterodimers, which permits the recognition of palindromic DNA-sequence motifs by adjacent DNA binding domains [2–4]. Specificity of NF-κB-mediated transcription is regulated in part by the combinatorial diversity arising from the five related NF-κB proteins, with each NF-κB dimer regulating both distinct and overlapping sets of genes due to subtle differences in their kB consensus DNA binding specificity [5]. Dimerization of NF-κB proteins, interaction with other transcriptional co-factors and DNA binding, is also regulated by extensive post-translational modification (PTM), with dynamic phosphorylation established as being critical for cellular function [6–12].

p105 (NFκB1) is one of the five NF-κB transcription factors and is commonly proteolysed to generate a functional p50 molecule lacking a transactivation domain [1]. The high basal levels of nuclear localised p50 homodimers in unstimulated cells are thus thought to act as repressors of transcription from κB promoters by competing for DNA binding with transcriptionally active NF-κB dimers, including the RelA:p50 heterodimer [13, 14]. Regulation of p50 by reversible phosphorylation is much less well understood than that of the p105 precursor [15–18], but appears to be a critical regulator of efficient p50 binding to DNA, and thus transcriptional repression. Phosphorylation of p50 in vitro by the catalytic subunit of protein kinase A (PKA_c_) has been reported to enhance its ability to bind DNA in a manner that is independent on its ability to dimerise [15, 16]. Mutational studies using phosphomimetic variants mapped this critical phosphorylation event to Ser337, and Ricciardi and colleagues demonstrated that this site is constitutively phosphorylated by PKA_c_ in unstimulated cells, contributing to κB transcriptional repression under basal (non-stimulated) conditions [15]. Mutation of two additional p50 Ser residues, Ser65 and Ser342, to Ala, was also reported to negatively influence the DNA binding ability of p50 [15]. However, regulated phosphorylation of these residues has not yet been demonstrated.

Mass spectrometry (MS) can be used in a variety of ways to elucidate information about all levels of protein structure, from primary to quaternary configurations. Exploitation of ‘native’ MS, where the analyte is transferred from solution into the gas-phase under gentle electrospray ionisation (ESI) conditions [19–21] from a volatile buffer such as ammonium acetate at physiological pH, allows non-covalent complexes to be interrogated. Native MS can thus be used to determine protein complex stoichiometry [22] or compare the relative dissociation constant (*K*_D_) of different ligands [23–25]. Many proteins and protein complexes largely retain their solution-phase conformation under native ESI conditions [26–28], thus their structure and the effect of protein modification and/or ligand binding on conformational dynamics and stability can be readily interrogated with gas-phase methods such as ion mobility spectrometry (IMS) [29–31] or infrared spectroscopy [32]. In IMS, ions are transported by an electric field through a drift cell filled with an inert gas such as helium or nitrogen, permitting separation of analyte ions based on their charge, mass and conformation. Consequently, the recorded drift time of ions through the IMS cell can be used to define their rotationally averaged collision cross section (CCS). Importantly, CCS values can be calculated for a given geometry from theoretical structures derived from density functional theory or molecular dynamic (MD) simulations using a variety of methods such as projection approximation (PA) [33], exact hard sphere scattering (EHSS) model [34], the trajectory method (TM) [35], projection superposition approximation (PSA) [36] or scattering on electron density isosurfaces (SEDI) [37]. Such computational strategies allow prediction of putative protein structures by comparison with experimentally derived CCS values derived using a variety of experimental-based structural approaches.

Here, we exploit standard MS-based phosphoproteomics in combination with native ion mobility-mass spectrometry (IM-MS) and molecular modelling to define the effects of p50 phosphorylation on dimerization and DNA binding. Using travelling wave-IMS (TW-IMS) and comparative molecular dynamics (MD) simulations, we demonstrate that the p50 homodimer is stabilised by the presence of its cognate DNA oligomer and define specific p50 phosphorylation sites as key potential regulators of either DNA binding or homo-dimerization.

## Experimental

### Protein Expression and Purification

Murine NF-κB p50 (39-364 wild-type; WT) was cloned into the pOPINM vector (OPPF) using the InFusion PCR cloning kit (Clonetech). S65D, S242D, S248D and S337D p50 mutations were generated by PCR site-directed mutagenesis from the WT p50 construct. Appropriate mutations were confirmed by DNA sequencing. All proteins were produced in BL21 (DE3) pLysS *E. coli* cells (Novagen) with expression induced with 0.5 mM IPTG for 3 h at 18 °C and purified with a 3C protease cleavable N-terminal His6-MBP-tag. Fusion proteins were first purified by affinity chromatography using amylose resin (NEB), and p50 subunits were cleaved from the immobilised affinity medium using 3C protease in 50 mM Tris (pH 7.4), 100 mM NaCl, 1 mM DTT, 10% (*v*/*v*) glycerol and 20 mM imidazole. 3C protease (purified as an N-terminal His6-tag fusion protein) was subsequently removed by immobilised metal affinity chromatography.

### In Vitro Phosphorylation, Digestion and LC-MS/MS Analysis

p50 protein (25 μg, 35-381, Enzo Scientific) in 10 mM TrisOAc was incubated at 37 °C for 2 h with 10 mM MgCl_2,_ 250 μM ATP, 1 mM DTT and 1 mM EGTA in the presence of 0.25 μg of either PKA_c_ [38] or 4.2 μg Chk1 (MRC PPU Reagents and Services, Dundee). Reactions were stopped by rapid buffer exchange into NH_4_OAc. For digestion, 1 μg of protein was denatured prior to digestion by addition of 1% Waters RapiGest at 80 °C for 10 min. Enzymatic digestion was performed at 37 °C overnight using 0.02 μg trypsin and stopped by addition of 0.5% TFA and incubation for 45 min at 37 °C. Phosphopeptides were enriched using TiO_2_ spin columns (GLSciences) as previously described [39]. LC-MS/MS analysis was performed on an Orbitrap Fusion Tribrid mass spectrometer (ThermoScientific), attached to an Ultimate 3000 nano system (Dionex). Peptides were loaded onto the trapping column (ThermoScientific, PepMap100, C18, 300 μm × 5 mm), using partial loop injection, for 7 min at a flow rate of 9 μL/min with 2% (*v*/*v*) MeCN 0.1% (*v*/*v*) TFA and then resolved on an analytical column (Easy-Spray C18 75 μm × 500 mm 2 μm bead diameter column) using a 30-min method from 96.2% A (0.1% FA) and 3.8% B (80% MeCN 19.9% H_2_O 0.1% FA) to 100% B at a flow rate of 300 nL min^−1^. A full scan mass spectrum was acquired (30K resolution at *m/z* 200) and data-dependent MS/MS analysis performed using a top speed approach (cycle time of 3 s), using HCD and EThcD fragmentation modes, with product ions being detected in the orbitrap (15K resolution).

### Native IM-MS and Collision-Induced Unfolding

A commercial TW-IMS instrument (Waters G2-Si) was utilised for native IM-MS. p50 was buffer exchanged into 100 mM NH_4_OAc using 10-kDa molecular cut-off spin filter columns (Amicon) and 1–3 μl of sample (typically 5 μM) was subjected to electrospray ionisation (ESI) at a voltage of 1.3–3 kV using a self-pulled nanospray tip. Sampling cone was set at 75 V. Trap pressure was adjusted to 5 × 10^−2^ mbar, He cell pressure was 4.53 mbar, IMS pressure was 2.78 mbar and transfer tube pressure was 5.18 × 10^−2^ mbar. IMS was performed using a travelling wave height of 29 V and a velocity of 650 m/s. Calibration of the TriWave device was performed as previously described [40, 41] using β-lactoglobulin A (Sigma L7880), avidin (Sigma A9275), transthyretin (Sigma P1742), concanavalin A (Sigma C2010) and serum albumin (Sigma P7656) as calibrants. Upon removing the time the ions spend in the time of flight mass spectrometer, a logarithmic plot of the ‘corrected drift time *t*’ versus the by charge *q* and reduced mass *μ* corrected CCS, the so-called reduced CCS was calculated and a straight line extrapolated in order to ascertain the slope, *m*, and intersection, *C*. The experimental ^TW^CCS_N2→He_ values (where the TW-IMS-determined drift times in a nitrogen atmosphere were converted to helium CCS values [42]) were finally calculated from measured drift times using Eq. 1:

1 $$ {}^{TW}C{CS}_{N_2\to He}=\frac{q}{\sqrt{\mu }}t{\hbox{'}}^m\bullet \exp (C) $$

CCSD values are defined as the full width at half maximum of the CCS distribution.

Collision-induced unfolding (CIU) was used to evaluate the transitional unfolding profiles of the protein and protein-DNA complexes. An individual charge state was isolated with the quadrupole mass filter and subjected to collisional activation in the trap region of the TriWave by application of gradually increasing collision energies. Contour plots representing the unfolding profile were produced with Origin 9.0.

### *K*_D_ Determination

p50 WT or single-point aspartic acid mutants (3 μM) were incubated with 0.2–6 μM of κB DNA oligomer 5′-CCCCCGGGGGCCCCCGGGGG-3′ (Sigma) in 300 mM NH_4_OAc for 5 min at room temperature (final volume 10 μL) prior to native (IM-)MS analysis. Multi-Gaussian fitting with Origin 9.0 was used to ascertain the peak areas of all charge states of both the unbound and DNA-bound p50. *K*_D_ values for DNA binding were determined by nonlinear peak fitting using Eq. 2:

2 $$ \frac{I\left(P\ast L\right)}{I(P)}=\frac{1}{2}\left(-1-\frac{{\left[P\right]}_0}{K_D}+\frac{{\left[L\right]}_0}{K_D}+\sqrt[]{4\frac{{\left[L\right]}_0}{K_D}+{\left(\frac{{\left[L\right]}_0}{K_D}-\frac{{\left[P\right]}_0}{K_D}-1\right)}^2}\right) $$

I(PL) and I(P) define the peak area of the DNA-bound protein complex and the unbound p50 protein dimer, respectively, [P]_0_ and [L]_0_ are the original protein and DNA concentrations [25].

### Molecular Modelling and CCS Calculation

ff14SB, OL15 and tip3p force fields implemented in AMBER16 [43] were used to simulate the effect of protein desolvation during ESI on the conformation of the DNA-bound and unbound forms of the p50 (39-350) homodimer (1.NFKB.pdb) [44]. The structure was neutralised by addition of Na^+^ ions and embedded into a water box containing approximately 35,000 water molecules. Geometry optimization for molecular dynamics (MD) simulation was performed utilising the steepest descent energy minimisation and conjugate gradient method. Molecular dynamic simulations were performed at 350 K for 2 ns, with 2 fs integration steps. Langevin dynamics were applied to regulate the temperature. The result of the 2 ns run was used as input for a further MD simulation in which the amount of water was reduced by ~ 10%. Upon completing 40 × 2 ns runs, a final 10-ns run was performed in the absence of solvent, using a charge state of 16+ for the p50 dimer and 18+ for the DNA-bound p50 dimer, those being the most dominant charge states observed. CCS values of all final MD structures were computed using Mobcal [34, 35]. RMSD values were calculated utilising CPPTRAJ implemented in AMBER [43].

## Results

### Chk1 Induces Extensive Phosphorylation of p50 In Vitro

Based on mutational analysis and in vitro protein kinase assays, it was previously reported that phosphorylation of p50 by PKA_c_ on Ser337 is essential for high-affinity DNA binding. However, the mechanism whereby pSer337 regulates DNA binding was not defined. Although a S337A p50 mutant exhibited dramatically reduced DNA binding ability in cells compared to the wild-type p50 protein, this site of modification is distal from the DNA binding domain, and the ability of S337A p50 to dimerise with p65/RelA was reported to be unaffected [16]. Moreover, the ability of p50 to be phosphorylated by PKA_c_, or other putative regulatory kinases, at sites distinct from Ser337 was not evaluated in side-by-side experiments.

**Figure 1:**
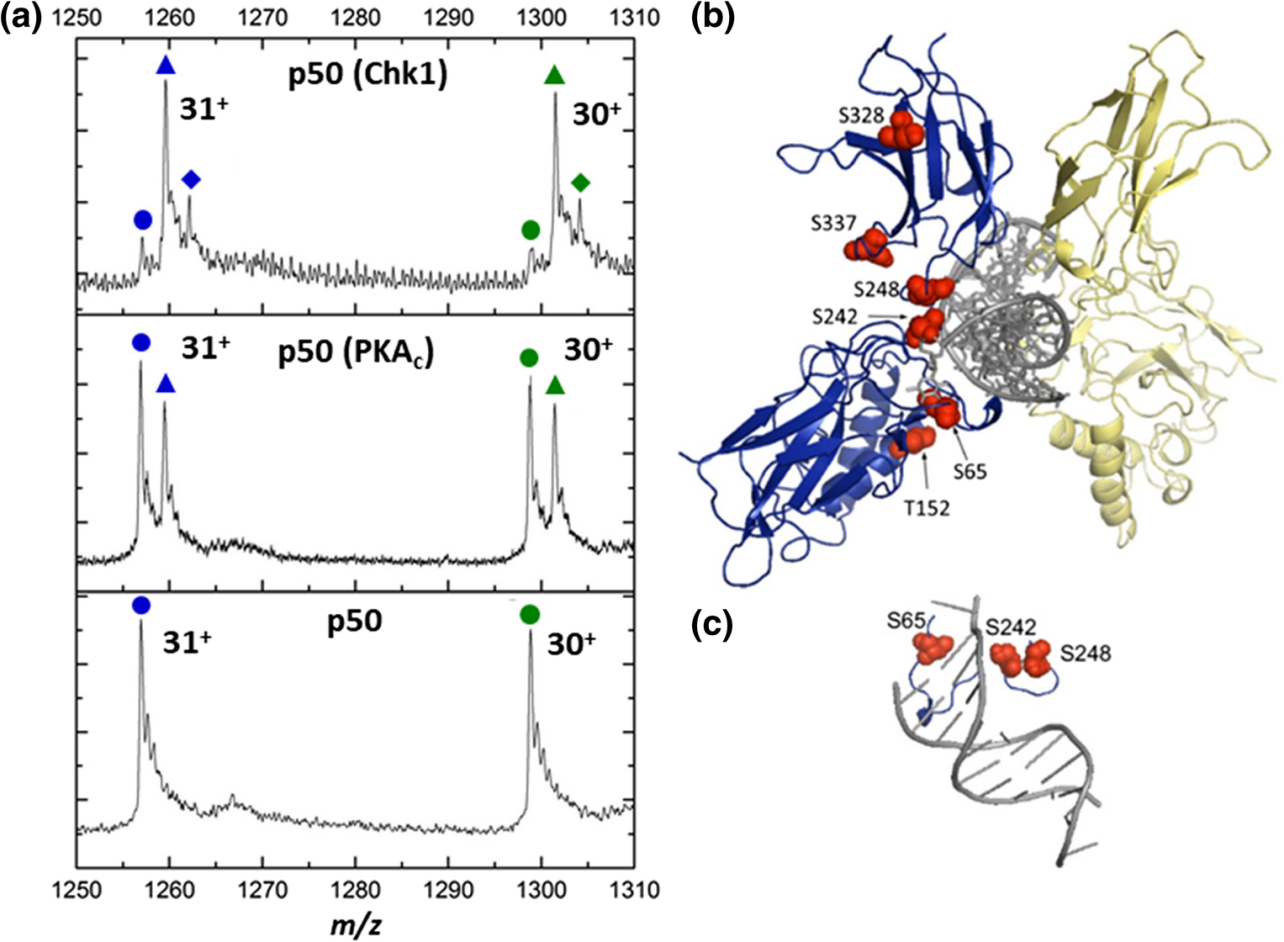
Multi-site phosphorylation of p50 by PKA_c_ and Chk1 is not combinatorial. (**a**) Intact mass spectra of p50 before (bottom) and after in vitro phosphorylation with PKA_c_ (middle) or Chk1 (top). Depicted are the 30+ (green) and 31+ (blue) charge states of the non-phosphorylated (circles), mono-phosphorylated (triangles) and di-phosphorylated (diamonds) forms of p50. (**b**) Identified sites of phosphorylation (red) mapped onto the X-ray crystal structure of the mouse p50:p50 homodimer bound to DNA (grey), PDB entry 1NFK. Individual p50 monomers are either in blue or yellow. Sites of phosphorylation are numbered according to the human sequence. Ser328 and Ser337 were identified as PKA_c_ phosphosites. All sites were phosphorylated by Chk1. (**c**) Loops 1 and 3 of p50 with kB DNA show direct interaction of the regions containing Ser65, Ser242 and Ser248 with the DNA

Using standard peptide-based tandem MS analysis, we recently reported PKA_c_-mediated phosphorylation of recombinant p50 (35-381) in the presence of p65/RelA at four sites in addition to Ser337, namely Ser223, Ser226, Ser236 and Thr263 [6]. To assess whether these phosphorylation sites are dependent on inclusion of RelA in the assay, and thus formation of a RelA:p50 heterodimer, we repeated in vitro phosphosite mapping using PKA_c_ with p50 alone. Under these conditions, only Ser328 and Ser337 were identified as PKA_c_-regulated phosphosites (Fig. 1, Supp. Figure 1), confirming previous observations that NF-κB proteins likely adopt different conformations dependent on their dimerization partners [6]. MS analysis of the intact phosphorylated p50 identified a single phosphate-carrying proteoform (in addition to the non-phosphorylated protein), with no evidence of a doubly phosphorylated species, suggesting that phosphorylation of p50 on Ser328 and Ser337 is likely to be mutually exclusive (Fig. 1a).

As well as cellular evidence for p50 regulation by PKA_c_ [18], Chk1 is known to play a major role in phosphorylation-mediated regulation of this transcription factor, inhibiting DNA binding via phosphorylation at Ser328 [45, 46]. Previous investigations have focused on phosphorylation of Ser328 by Chk1, even though p50 contains a number of other conserved Chk1 consensus sites. Consistently, we identified a total of six in vitro Chk1 phosphorylation sites on p50 (35-381) using MS-based phosphopeptide mapping (Supp. Figure 1), including the previously reported Ser328 site [46], the overlapping PKA_c_ site at Ser337, and four novel sites at Ser65, Thr152, Ser242 and Ser248. Chk1 phosphorylation of p50, at least in vitro, is thus much more extensive than previously supposed. Analysis of the intact Chk1-phosphorylated p50 reveals a predominant singly phosphorylated species, as well as a doubly phosphorylated form, with relatively low levels of non-phosphorylated p50 (Fig. 1a). Analogous to the PKA_c_-phosphorylated p50, the six sites modified by Chk1 are thus unlikely to be stoichiometrically combinatorial, rather, p50 is modified at specific (discrete) combinations of amino acids.

Of the 10 phosphosites that we identified in total on p50 (with or without RelA), only two, Thr152 and Ser226, are not completely conserved in model vertebrates (Supp. Figure 2). Thr152 is changed to Ile in *Xenopus laevis* p50, although it is conserved as a Thr in all other species examined. Ser226 was absent in both frog and chicken p50 sequences. Considering the position of the six PKA_c_ and Chk1 phosphosites identified in the absence of p65 in the p50 homodimer structure (PDB entry 1NFK [44]), a number of potential roles for phosphorylation might be hypothesised. Ser242 and Ser248 (mouse Ser240 and Ser246 respectively) both lie in the linker region (L3) between the two distinct domains of p50 (Fig. 1b, c). Phosphorylation of one or both of these residues in this linker region, which adopts a well-defined structure that can fit into the major groove of the DNA substrate, is thus likely to have a significant effect on DNA binding ability of p50. In particular, Ser242 lies adjacent to a key Lys residue at position 243 (mouse Lys241), which directly interacts with the DNA backbone. Consequently, we hypothesised that Ser242 phosphorylation is likely to disrupt p50 DNA binding. Similarly, Ser65 (mouse Ser63) lies downstream of a five residue cluster (RxRYxCExx**S)** located in L1, another loop that makes direct contacts with the κB DNA. Even though phosphorylation of both Ser328 and Ser337 has been shown to influence the ability of p50 to bind DNA, both are localised to the second domain, distal from the DNA-binding region, suggesting a gross conformational change of domain 1 with respect to domain 2 and the DNA-protein interface, rather than a direct effect of phosphorylation of these residues on the ability to bind DNA.

### Phosphorylation of p50 by Chk1 Destabilises Dimerization

**Figure 2:**
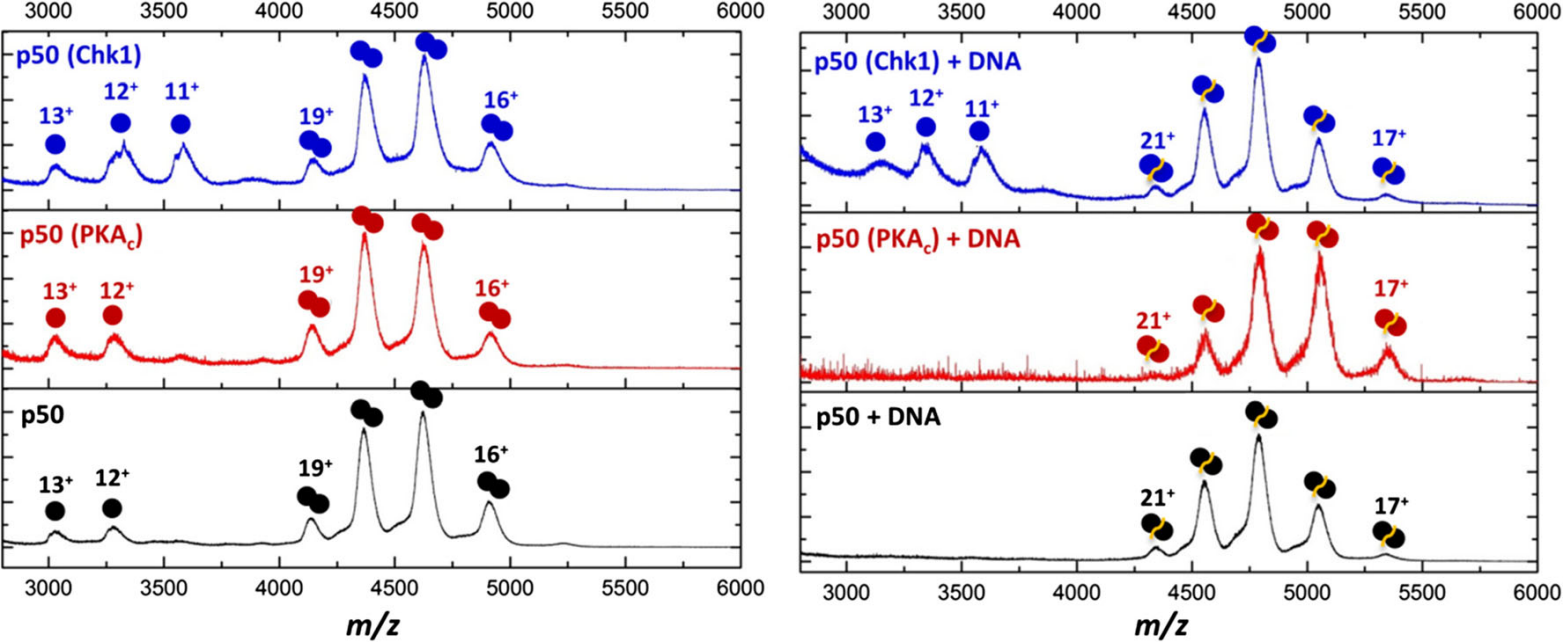
Phosphorylation of p50 regulates dimerization. Native mass spectra of p50 before (bottom) or after phosphorylation with either PKA_c_ (middle, red) or Chk1 (top, blue), in the absence (left) or presence (right) of the p50 kB DNA oligomer. Charge states are indicated

To assess the effect of p50 phosphorylation on its ability to dimerise and bind DNA, we analysed p50 (35-381) by nano-electrospray ionisation (nESI)-MS under non-denaturing ‘native’ MS conditions, before and after in vitro phosphorylation with either PKA_c_ or Chk1. As expected, intact non-phosphorylated p50 was preferentially observed as a dimer with only a small amount of monomer present (Fig. 2). Upon phosphorylation with either protein kinase, there was a small but consistent increase in the relative abundance of the p50 monomer (observed charge states of 11+ to 13+) with respect to the p50 homodimer (observed charge states of 16+ to 19+), demonstrating phosphorylation-mediated destabilisation of the homodimeric protein.

Inclusion of a DNA oligomer designed to match the κB consensus sequence for the p50 homodimer (5′-CCCCCGGGGGCCCCCCGGGGG-3′) revealed a stabilising effect of DNA binding upon dimer formation. No residual monomer was observed for the non-phosphorylated p50 in the presence of the κB DNA, with stoichiometric formation of the p50:p50:DNA complex. A similar stabilising effect was also seen for PKA_c_-phosphorylated p50, with no monomeric p50 observed and stoichiometric DNA:protein complex formed. In contrast, although the Chk1-phosphorylated p50 homodimer stoichiometrically bound the κB DNA, dimer formation was not enhanced under these conditions (Fig. 2). Chk1 phosphorylation of p50 thus appears to actively disrupt homo-dimerization, irrespective of effects on DNA binding.

To further evaluate the structural effects of p50 phosphorylation, we used ion mobility-MS (IM-MS) to compare p50 conformation and structural dynamics following treatment with either PKA_c_ or Chk1 (see supplementary information; Supp. Figure 3). The rotationally averaged collision cross section (^TW^CCS_N2→He_) of the p50 homodimer was determined as 44.3 nm^2^, relatively independent of the corresponding charge state (16+ to 18+) (Supp. Figure 3). Although subtle differences were observed in the p50 ^TW^CCS_N2→He_ distribution (^TW^CCSD_N2→He_) upon phosphorylation with PKA_c_, particularly for the 16+ and 17+ charge states, there was no statistically significant change in the absolute ^TW^CCS_N2→He_ value after phosphorylation with either PKA_c_ or Chk1 (Supp. Figure 3).

Interestingly, there was a small but reproducible 1.5% decrease in the ^TW^CCS_N2→He_ values of the DNA-bound p50 dimer with decreasing charge state (20+ to 18+), revealing slight compaction of the complex. Moreover, the asymmetric CCS profile of the WT p50:p50:DNA complex is indicative of the presence of two unresolved conformers with charge state averaged ^TW^CCS_N2→He_ values of 51.1 and 53.3 nm^2^ for the unmodified p50-DNA dimer. Of note, the relative proportion of the more compact conformer increased with a reduction in charge state, suggesting gas-phase conformational contraction. Comparable results were also observed following native IM-MS analysis of p50 (39-364) (Supp. Figure 4).

### Ser337 Phosphomimetic Disrupts p50 Homodimer Formation

**Figure 3:**
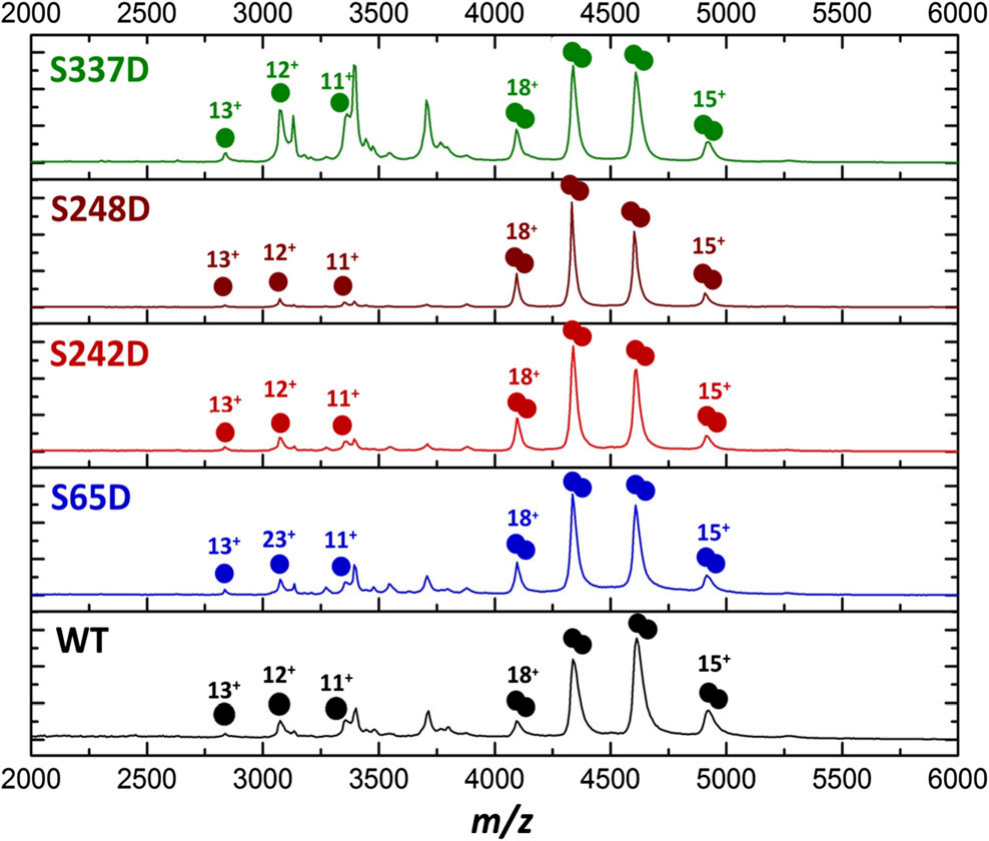
Ser337 phosphomimetic disrupts dimerization of p50. Native mass spectra of p50 (39-364) wild-type (WT, black), S65D (blue), S242D (red), S248D (brown) and S337D (green) phosphomimetic versions showing p50 monomer and dimer. Non-assigned peaks derive from the cleaved contaminating MBP expression tag. Charge states are indicated

To evaluate which of the site-specific, but sub-stoichiometric PKA_c_- or Chk1-mediated phosphorylation events were responsible for the observed disruption of dimerization, we expressed Ser → Asp (potential phosphomimetic) versions of p50 (39-364) for sites predicted to influence either dimer formation or DNA binding: Ser65, Ser242, Ser248 lie in close contact with the DNA, and Ser337 is located in the dimerization region and might impart allosteric regulation of dimerization upon phosphorylation. S65D, S242D, S248D and S337D p50 were analysed by native IM-MS alongside WT p50 (Fig. 3), using multi-Gaussian fitting to evaluate the peak areas and calculate the monomer:dimer ratio for each species.

Akin to WT p50, the dominant species of all S → D p50 protein mass spectra were dimers (Fig. 3). Little, or no, difference was observed in the monomer:dimer ratios for S65D and S242D p50 (Fig. 3, Table 1). However, there was a 2.4-fold relative increase in monomeric S337D p50 compared to the non-phosphorylated WT p50. The ability of S337D to disrupt p50 dimerization was further confirmed by size exclusion chromatography (SEC), which revealed a ~ 5-fold relative increase in monomeric S337D p50 based on protein staining and densitometry (Supp. Figure 5). Together, these findings support our initial prediction that Ser337 phosphorylation plays a significant role in controlling (disrupting) p50 dimerization.

**Table 1:** Effect of p50 phosphomimetic variants on protein dimerization and DNA binding. Percentage peak areas of monomers and dimers for p50 WT, S65D, S242D, S248D and S337D and the mutants. DNA binding dissociation constants *K*_D_, and the relative DNA binding affinity with respect to WT p50, are also presented for each p50 variant

	Monomer	Dimer	*K*_D_ value for DNA binding (nM)	Relative DNA binding affinity
WT	10.5%	89.5%	37 ± 7	100%
S65D	9.4%	90.6%	140 ± 16	26.4%
S242D	12.4%	87.6%	424 ± 78	8.7%
S248D	5.8%	94.2%	112 ± 11	33.0%
S337D	25.2%	74.8%	111 ± 15	33.3%

**Figure 4:**
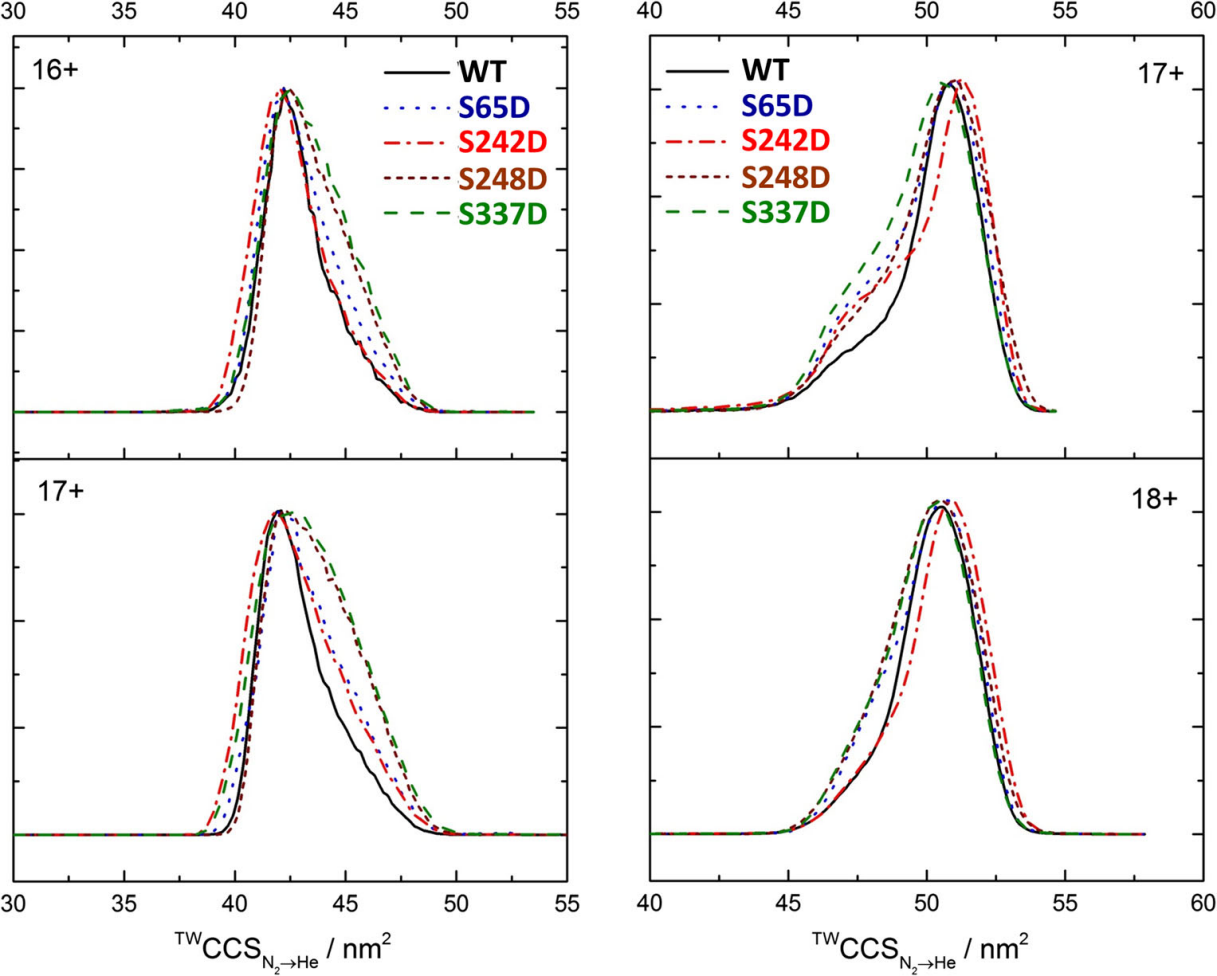
Phosphomimetic versions of p50 exhibit increased conformational flexibility compared to WT p50. ^TW^CCS_N2→He_ distributions of wild-type (WT) p50 (39-364) (black) alongside p50 S65D (blue), S242D (red), S248D (brown) and S337D (green) in the absence (left) or presence (right) of DNA

IM-MS analysis of the p50 protein variants revealed a peak maximum ^TW^CCS_N2→He_ of ~ 42.0–42.5 nm^2^ suggesting that the dominant dimer conformation is highly similar for all species analysed (Fig. 4). However, a notable increase in conformational flexibility was observed for all mutants compared with WT p50. In particular, the CCSD values for S248D and S337D are 4.6 and 5.0 nm^2^, respectively, compared with a CCSD value of just 2.9 nm^2^ for WT p50 (Fig. 4).

### Phosphomimetic Version of the Chk1-Mediated p50 Phosphosite Ser242 Destabilises DNA Binding

To evaluate the effect of modification of S248 and S337 on DNA binding, we employed the titration method and native MS to determine the dissociation constants (*K*_D_) for DNA binding for each of the p50 protein variants (Fig. 4; Table 1). As expected, the unphosphorylated WT p50 homodimer exhibited a relatively high affinity for DNA in this assay, with a *K*_D_ value of < 40 nM, similar to that previously reported for both p65 homodimers and p65/p50 heterodimers [47]. All the aspartic acid mutants analysed exhibited significantly higher *K*_D_ values than those observed for WT p50, indicating a reduction in DNA binding affinity. Specifically, S337D p50 exhibited a 3-fold higher dissociation constant than WT p50, with a measured *K*_D_ of ~ 110 nM, compared with a *K*_D_ of 37 nM for WT p50 under the same conditions. This increase in relative *K*_D_ is likely attributed to the reduction in the ability of this phosphomimetic variant to dimerise (Table 1), a prior requirement for DNA binding.

In agreement with our hypothesis, mimicking the Chk1-mediated p50 phosphorylation site at S242 resulted in an order of magnitude decrease in DNA binding affinity. Replacement of Ser242 with a negatively charged Asp group is predicted to disrupt the direct electrostatic interaction of Lys243 with the phosphate backbone of the DNA. Interestingly, the consistently earlier arrival time distribution (ATD) of S242D when compared to that of WT p50 observed in the absence of DNA suggests that this protein can adopt conformations that are likely to be more compact than WT p50 (Fig. 4). In contrast, upon DNA binding, the ^TW^CCS_N2→He_ of S242D is consistently larger than the WT dimeric complex, indicative of a more open conformation.

**Figure 5:**
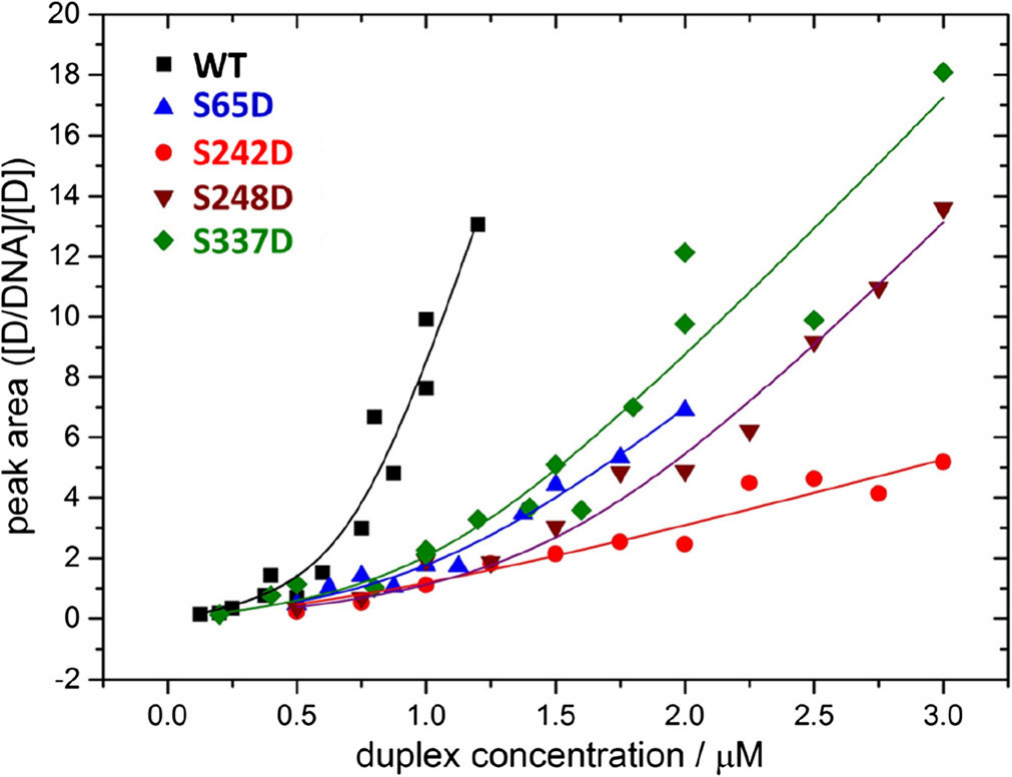
DNA binding of p50 S242D is significantly disrupted. Native MS and DNA titration was used to calculate *K*_D_ for DNA binding for each of the p50 homodimers: WT (black), S65D (blue), S242D (red), S248D (brown) and S337D (green)

Mutation of the other two identified p50 phosphosites in the DNA binding interface, S65 and S248 (Fig. 1b), resulted in a 3.8- and 3.0-fold increase in *K*_D_ values, respectively, compared with the WT protein (Fig. 5), implicating roles for phosphorylation of S65 and S248 in negatively regulating DNA binding of the p50 homodimer. However, ATDs of these two p50 variants are distinct, both in the absence and presence of DNA, indicative of different effects on gross conformation. The similarity of the ATDs of S248D and S337D, which lie in the L3 linker region of p50 and the dimerization domain respectively (Fig. 1), suggests that both of these phosphomimetic mutations similarly alter the relative position of the two domains of p50.

### Comparison with Theoretical Modelling

**Figure 6:**
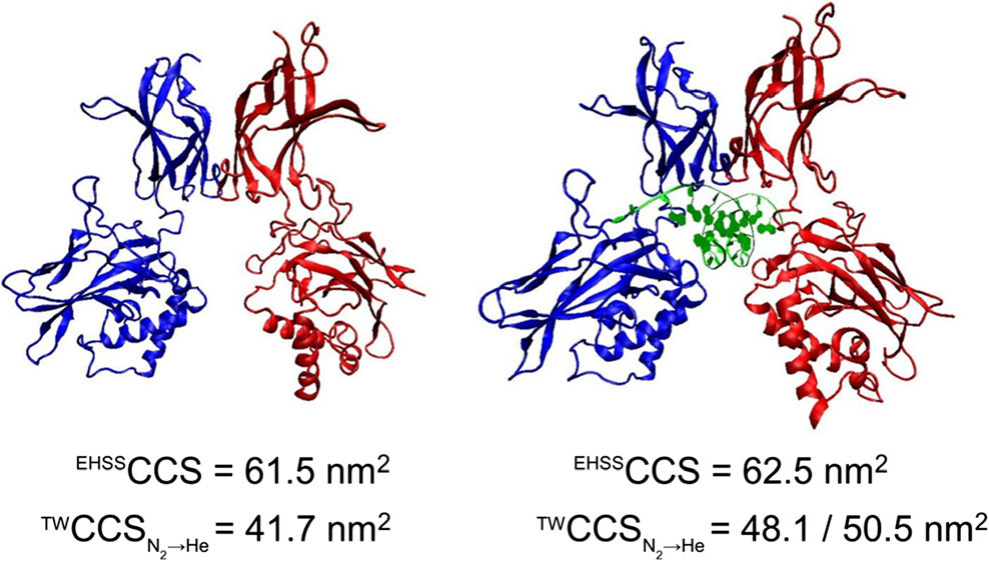
Gas-phase structure of the p50 homodimer is stabilised in the presence of DNA. Mobcal was used to determine the theoretical (^EHSS^CCS) value of the p50 dimer using the exact hard sphere scattering (EHSS) model, in the absence (left) and presence (right) of DNA from the X-ray structure, for comparison with experimentally determined cross section values (^TW^CCS_N2→He_)

To further interrogate the observed differences in p50 conformers in the absence and presence of DNA, and to assess whether the condensed phase structure is maintained in the gas-phase upon ‘native’ ESI, we compared the experimentally determined cross sections (^TW^CCS_N2 → He_) with theoretically calculated (^EHSS^CCS) values based on the exact hard sphere scattering modal (EHSS) implemented in Mobcal (Fig. 6).

Both the unbound and DNA-bound forms of the p50 (WT) dimer possess very similar ^EHSS^CCS of 61.5 and 62.5 nm^2^, which is perhaps not surprising given that they only differ by the small central cavity which accommodates the helical DNA. In contrast and as previously observed, there was a significant difference in the ^TW^CCS_N2→He_ of these two complexes. The DNA-bound protein dimer exhibited two major conformers of 48.1 and 50.5 nm^2^ (Supp. Figure 4). Crucially, the ^TW^CCS_N2→He_ is consistently smaller than the ^EHSS^CCS, suggesting ‘contraction’ from the condensed phase structure, consistent with many other reports [48–51]. While the difference between the ^EHSS^CCS and ^TW^CCS_N2→He_ for the DNA-bound p50 dimer was between 19 and 27% (conformer-dependent), this increased to 32% for the non DNA-bound complex, indicating a more extensive contraction in the absence of DNA in the central core.

**Figure 7:**
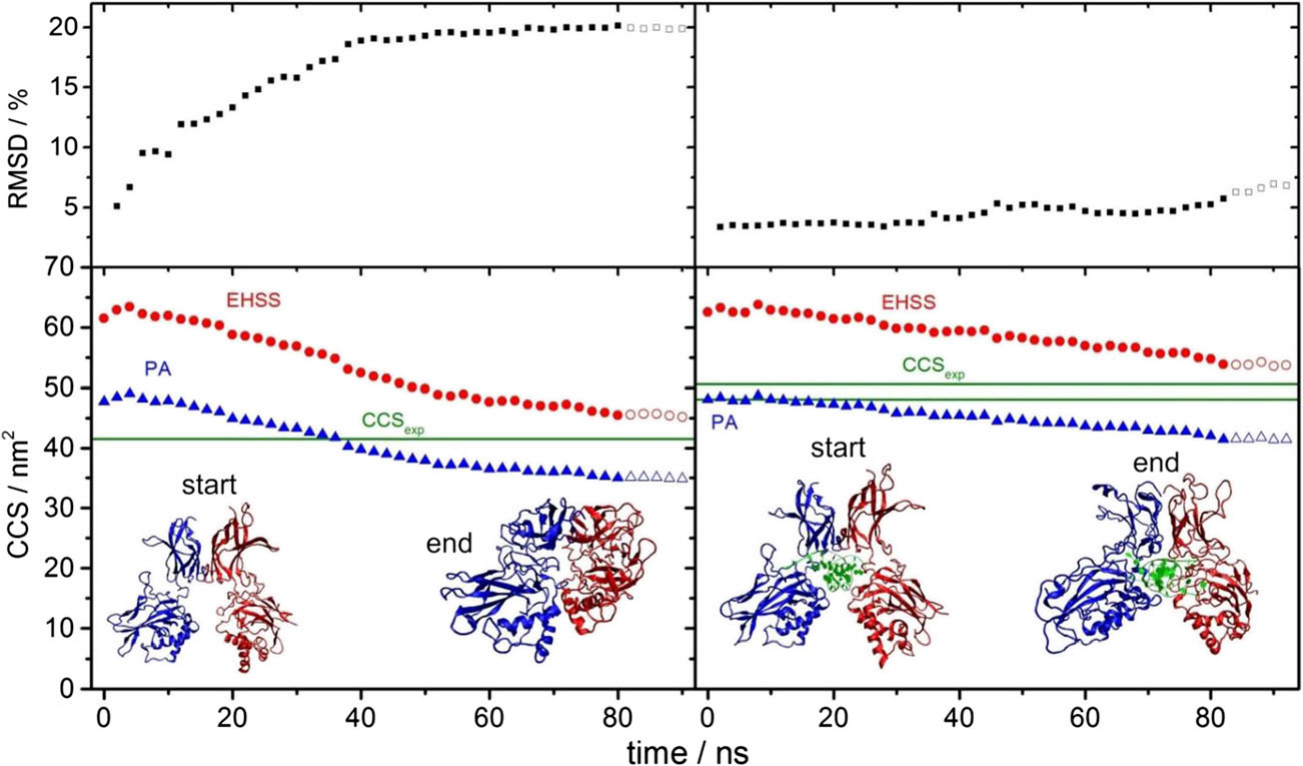
Solvent evaporation during electrospray ionisation results in collapse of the p50 dimer which is partially stabilised in the presence of DNA. Simulation of the evaporation process of the unbound (left) and DNA-bound (right) p50 dimer. Each data point represents the final CCS value calculated using either the projection approximation (PA, blue) or the exact hard sphere scattering (EHSS, red) model of a 2-ns run upon removing 10% of the solvent. Empty symbols correspond to CCS values of the final gas-phase simulation. Root mean square deviation (RMSD) values of each final structure are displayed (top). Green lines exemplify the experimentally determined ^TW^CCS_N2→He_ values (CCS_exp_), with two predominant conformers being defined for the DNA-bound p50 dimer

To better understand the reasons for this contraction, we investigated the evaporation process during ESI, monitoring structural changes during transfer to the gas phase, and performing a molecular dynamics simulation over 80 ns (Fig. 7). Root mean square deviation (RMSD) as well as ^EHSS^CCS values were calculated for the final structure of each 2-ns run after removing ~ 10% of the solvent molecules. The RMSD values of the unbound WT p50 dimer as a function of the simulation time reveal an increase of up to 20%, implying a significant conformational change upon desolvation.

Using the projection approximation (PA) and the exact hard sphere scattering (EHSS) models, the theoretically calculated CCS values of the p50 dimer exhibited conformational contraction, with the ^TW^CCS_N2→He_ fitting between two theoretical values of the final gas-phase simulation. Indeed, the ^TW^CCS_N2→He_ was only 8% smaller than the more exact EHSS value (Fig. 7), which is similar to the differences observed in other studies [50]. By considering the final modelled gas-phase structure, it is apparent that the conformational change in the p50 dimer induced upon evaporation is mostly related to the removal of the inner cavity.

The final gas-phase structure of the DNA-bound p50 dimer exhibited a much lower RMSD value (6.7%), implying a more conservative structural change compared to the unbound dimer. Again, the two experimentally determined CCS values are located between the theoretical PA and EHSS values. Although we cannot assign structures for the two experimentally observed conformations, ^TW^CCS_N2→He_ for the more abundant ‘open’ conformer is only 5.5% smaller than the ^EHSS^CCS value. Consequently, it is likely that the final MD structure is similar to the dominant experimental gas-phase conformer. The major changes in this final simulated gas-phase structure occur in the outer loop (surface) regions, indicating that the presence of DNA in the central cavity of the p50 dimer likely functions to stabilise the structure and prevent collapse of the inner core during ESI-IM-MS.

Collision-induced unfolding of a single common charge state (17+) was used to assess the relative stability of these two complexes in more detail (Supp. Figure 6). A three-step unfolding profile is observed for the non DNA-bound p50 dimer, occurring at collision voltages (CVs) of 40, 48 and 58 V. For the DNA-bound dimer, initial collisional activation induces marginal structural contraction (decreased CCS), before collision-mediated elongation. This structural compaction event prior to unfolding is similar to that reported previously for the dimeric p53 transcription factor [52]. For the DNA-bound p50 dimer, the first transition to a more unfolded conformation takes place at a CV of 59 V, 19 V higher than that required to start mediating unfolding of the non DNA-bound dimer. These results underpin our findings that DNA binding helps to stabilise the structure of the p50 dimer, although interestingly the unfolded highly activated structures for both complexes have similar CCS values, suggesting a similar unfolded conformation.

## Conclusion

A central goal of this study was to gain insight into the potential mechanisms of phosphorylation-mediated transcriptional regulation through the NF-κB transcription factor p50, by exploring the effects of specific phosphorylation events on its ability to dimerize and interact with DNA. The two known p50 regulatory protein kinases under investigation, PKA_c_ and Chk1, were shown to phosphorylate p50 in vitro on two and six sites respectively by LC-MS. Native MS analysis of p50 in the absence and presence of the kB DNA oligomer revealed significant differences in protein dimerization after phosphorylation with either enzyme, with the change in dimer to monomer ratio suggesting that at least one of the phosphorylation sites was responsible for regulating p50 dimerization. By expressing and evaluating site-specific acidic ‘phosphomimetic’ versions of p50, where established sites of phosphorylation were mutated to aspartic acid, we were able to define a role for Ser337 (phosphorylated by both PKA_c_ and Chk1) as a critical regulator of p50 homo-dimerization. These findings are in contrast to a previous report, which implied a direct effect of PKA_c_-mediated phosphorylation at Ser337 on the ability of p50:p65 heterodimers to bind DNA, in the absence of an effect on p50 dimerization [15].

Based on our observations of an order of magnitude decrease in the relative DNA binding affinity of a S242D p50 mutant, we determined that the Chk1 (but not PKA_c_)-mediated phosphorylation of Ser242 is a key regulator of p50 homodimer DNA binding. Ser242 maps to the DNA binding interface of p50 and lies immediately *N*-terminal to a critical Lys residue that drives a direct interaction with the DNA phosphate backbone. Phosphorylation of Ser242 is therefore likely to serve as a mechanism to directly disrupt this interaction, perhaps upon Chk1 activation in cells subject to genotoxic stress.

Finally, comparison of our experimentally derived ^TW^CCS_N2→He_ values (CCS_exp_) with theoretically calculated values (CCS_the_) derived from the p50 dimer X-ray structure confirmed that although there was little difference in the CCS_the_ for the p50 dimer upon addition of DNA, the CCS_exp_ values for these complexes were markedly differed. By performing molecular dynamics simulations, we uncovered desolvation-mediated structural contraction of the p50 homodimer during ESI, which was stabilised by the presence of DNA in the central cavity. The final modelled gas-phase structures, whose EHSS values are highly similar to the CCS_exp_ of the more abundant elongated conformer, suggest only minor conformational changes on the surface of the protein complex when compared with the X-ray crystal structure. This stabilising effect of DNA on the structure of the dimeric p50 transcription factor was further confirmed by examining the collision-induced unfolding conformational profile, and again demonstrates how native MS can be used to derive structural information for this important class of DNA-binding proteins [52, 53].

## Electronic Supplementary Material

ESM 1 (DOCX 1008 kb)

## References

[1] Hayden MS, Ghosh S (2012). NF-kappa B, the first quarter-century: remarkable progress and outstanding questions. Genes Dev..

[2] Abel T, Maniatis T (1989). Gene regulation. Action of leucine zippers. Nature.

[3] Busch SJ, Sassone-Corsi P (1990). Dimers, leucine zippers and DNA-binding domains. Trends Genet..

[4] Kerppola TK, Curran T (1991). Fos-Jun heterodimers and Jun homodimers bend DNA in opposite orientations: implications for transcription factor cooperativity. Cell.

[5] Fujita T, Nolan GP, Ghosh S, Baltimore D (1992). Independent modes of transcriptional activation by the p50 and p65 subunits of NF-kappa B. Genes Dev..

[6] Lanucara, F., Lam, C., Mann, J., Monie, T.P., Colombo, S.A., Holman, S.W., Boyd, J., Dange, M.C., Mann, D.A., White, M.R., Eyers, C.E.: Dynamic phosphorylation of RelA on Ser42 and Ser45 in response to TNFalpha stimulation regulates DNA binding and transcription. Open Biol. **6**, (2016) 10.1098/rsob.160055 PMC4967822 27466442

[7] Huang B, Yang XD, Lamb A, Chen LF (2010). Posttranslational modifications of NF-kappaB: another layer of regulation for NF-kappaB signaling pathway. Cell. Signal..

[8] Lu T, Stark GR (2015). NF-kappaB: regulation by methylation. Cancer Res.

[9] Perkins ND (2006). Post-translational modifications regulating the activity and function of the nuclear factor kappa B pathway. Oncogene.

[10] Viatour P, Merville MP, Bours V, Chariot A (2005). Phosphorylation of NF-kappaB and IkappaB proteins: implications in cancer and inflammation. Trends Biochem. Sci..

[11] Chen J, Chen ZJ (2013). Regulation of NF-kappaB by ubiquitination. Curr. Opin. Immunol..

[12] Li H, Wittwer T, Weber A, Schneider H, Moreno R, Maine GN, Kracht M, Schmitz ML, Burstein E (2012). Regulation of NF-kappaB activity by competition between RelA acetylation and ubiquitination. Oncogene.

[13] Cheng CS, Feldman KE, Lee J, Verma S, Huang DB, Huynh K, Chang M, Ponomarenko JV, Sun SC, Benedict CA, Ghosh G, Hoffmann A (2011). The specificity of innate immune responses is enforced by repression of interferon response elements by NF-kappaB p50. Sci Signal.

[14] Zhong H, May MJ, Jimi E, Ghosh S (2002). The phosphorylation status of nuclear NF-kappa B determines its association with CBP/p300 or HDAC-1. Mol. Cell.

[15] Hou S, Guan H, Ricciardi RP (2003). Phosphorylation of serine 337 of NF-kappaB p50 is critical for DNA binding. J. Biol. Chem..

[16] Guan H, Hou S, Ricciardi RP (2005). DNA binding of repressor nuclear factor-kappaB p50/p50 depends on phosphorylation of Ser337 by the protein kinase A catalytic subunit. J. Biol. Chem..

[17] Lang V, Janzen J, Fischer GZ, Soneji Y, Beinke S, Salmeron A, Allen H, Hay RT, Ben-Neriah Y, Ley SC (2003). betaTrCP-mediated proteolysis of NF-kappaB1 p105 requires phosphorylation of p105 serines 927 and 932. Mol. Cell. Biol..

[18] Salmeron A, Janzen J, Soneji Y, Bump N, Kamens J, Allen H, Ley SC (2001). Direct phosphorylation of NF-kappaB1 p105 by the IkappaB kinase complex on serine 927 is essential for signal-induced p105 proteolysis. J. Biol. Chem..

[19] Leney AC, Heck AJ (2017). Native mass spectrometry: what is in the name?. J. Am. Soc. Mass Spectrom..

[20] Rosati S, Rose RJ, Thompson NJ, van Duijn E, Damoc E, Denisov E, Makarov A, Heck AJ (2012). Exploring an orbitrap analyzer for the characterization of intact antibodies by native mass spectrometry. Angew Chem Int Ed Engl.

[21] Sharon M, Robinson CV (2007). The role of mass spectrometry in structure elucidation of dynamic protein complexes. Annu. Rev. Biochem..

[22] Hernandez H, Robinson CV (2007). Determining the stoichiometry and interactions of macromolecular assemblies from mass spectrometry. Nat. Protoc..

[23] Daniel JM, McCombie G, Wendt S, Zenobi R (2003). Mass spectrometric determination of association constants of adenylate kinase with two noncovalent inhibitors. J. Am. Soc. Mass Spectrom..

[24] Cubrilovic D, Biela A, Sielaff F, Steinmetzer T, Klebe G, Zenobi R (2012). Quantifying protein-ligand binding constants using electrospray ionization mass spectrometry: a systematic binding affinity study of a series of hydrophobically modified trypsin inhibitors. J. Am. Soc. Mass Spectrom..

[25] Eyers CE, Vonderach M, Ferries S, Jeacock K, Eyers PA (2018). Understanding protein-drug interactions using ion mobility-mass spectrometry. Curr. Opin. Chem. Biol..

[26] Jurneczko E, Barran PE (2011). How useful is ion mobility mass spectrometry for structural biology? The relationship between protein crystal structures and their collision cross sections in the gas phase. Analyst.

[27] Breuker K, McLafferty FW (2008). Stepwise evolution of protein native structure with electrospray into the gas phase, 10(-12) to 10(2) s. Proc. Natl. Acad. Sci. U. S. A..

[28] Scarff CA, Thalassinos K, Hilton GR, Scrivens JH (2008). Travelling wave ion mobility mass spectrometry studies of protein structure: biological significance and comparison with X-ray crystallography and nuclear magnetic resonance spectroscopy measurements. Rapid Commun. Mass Spectrom..

[29] Mason, E.A., Schamp Jr., H.W.: Mobility of gaseous ions in weak electric fields. Ann. Phys. **4**, 233–270 (1958)

[30] Lanucara F, Holman SW, Gray CJ, Eyers CE (2014). The power of ion mobility-mass spectrometry for structural characterization and the study of conformational dynamics. Nat. Chem..

[31] Bowers MT (2014). Ion mobility spectrometry: a personal view of its development at UCSB. Int. J. Mass Spectrom..

[32] Ujma J, Kopysov V, Nagornova NS, Migas LG, Lizio MG, Blanch EW, MacPhee C, Boyarkin OV, Barran PE (2018). Initial steps of amyloidogenic peptide assembly revealed by cold-ion spectroscopy. Angew Chem Int Ed Engl.

[33] Marklund EG, Degiacomi MT, Robinson CV, Baldwin AJ, Benesch JL (2015). Collision cross sections for structural proteomics. Structure.

[34] Shvartsburg AA, Jarrold MF (1996). An exact hard-spheres scattering model for the mobilities of polyatomic ions. Chem. Phys. Lett..

[35] Mesleh MF, Hunter JM, Shvartsburg AA, Schatz GC, Jarrold MF (1996). Structural information from ion mobility measurements: effects of the long-range potential. J Phys Chem-Us.

[36] Bleiholder C, Wyttenbach T, Bowers MT (2011). A novel projection approximation algorithm for the fast and accurate computation of molecular collision cross sections (I) method. Int J Mass Spectrom.

[37] Shvartsburg AA, Liu B, Jarrold MF, Ho KM (2000). Modeling ionic mobilities by scattering on electronic density isosurfaces: application to silicon cluster anions. J. Chem. Phys..

[38] Byrne DP, Vonderach M, Ferries S, Brownridge PJ, Eyers CE, Eyers PA (2016). cAMP-dependent protein kinase (PKA) complexes probed by complementary differential scanning fluorimetry and ion mobility-mass spectrometry. Biochem. J..

[39] Ferries S, Perkins S, Brownridge PJ, Campbell A, Eyers PA, Jones AR, Eyers CE (2017). Evaluation of parameters for confident phosphorylation site localization using an orbitrap fusion tribrid mass spectrometer. J. Proteome Res..

[40] Smith DP, Knapman TW, Campuzano I, Malham RW, Berryman JT, Radford SE, Ashcroft AE (2009). Deciphering drift time measurements from travelling wave ion mobility spectrometry-mass spectrometry studies. Eur J Mass Spectrom.

[41] Ruotolo BT, Benesch JLP, Sandercock AM, Hyung SJ, Robinson CV (2008). Ion mobility-mass spectrometry analysis of large protein complexes. Nat. Protoc..

[42] Surman AJ, Robbins PJ, Ujma J, Zheng Q, Barran PE, Cronin L (2016). Sizing and discovery of nanosized polyoxometalate clusters by mass spectrometry. J. Am. Chem. Soc..

[43] Case, D.A., Cerutti, D.S., Cheatham, T.E., Darden, T.A., Duke, R.E., Giese, T.J., Gohlke, H., Goetz, A.W., Greene, D., Homeyer, N., Izadi, S., Kovalenko, A., Lee, T.S., LeGrand, S., Li, P., Lin, C., Liu, J., Luchko, T., Luo, R., Mermelstein, D., Merz, K.M., Monard, G., Nguyen, H., Omelyan, I., Onufriev, A., Pan, F., Qi, R., Roe, D.R., Roitberg, A., Sagui, C., Simmerling, C.L., Botello-Smith, W.M., Swails, J., Walker, R.C., Wang, J., Wolf, R.M., Wu, X., Xiao, L., York, D.M., Kollman, P.A.: AMBER 2017. University of California, San Francisco, (2017)

[44] Ghosh G, van Duyne G, Ghosh S, Sigler PB (1995). Structure of NF-kappa B p50 homodimer bound to a kappa B site. Nature.

[45] Schmitt AM, Crawley CD, Kang S, Raleigh DR, Yu X, Wahlstrom JS, Voce DJ, Darga TE, Weichselbaum RR, Yamini B (2011). p50 (NF-kappaB1) is an effector protein in the cytotoxic response to DNA methylation damage. Mol. Cell.

[46] Crawley CD, Raleigh DR, Kang S, Voce DJ, Schmitt AM, Weichselbaum RR, Yamini B (2013). DNA damage-induced cytotoxicity is mediated by the cooperative interaction of phospho-NF-kappaB p50 and a single nucleotide in the kappaB-site. Nucleic Acids Res..

[47] Bergqvist S, Alverdi V, Mengel B, Hoffmann A, Ghosh G, Komives EA (2009). Kinetic enhancement of NF-kappaBxDNA dissociation by IkappaBalpha. Proc. Natl. Acad. Sci. U. S. A..

[48] Hall Z, Politis A, Bush MF, Smith LJ, Robinson CV (2012). Charge-state dependent compaction and dissociation of protein complexes: insights from ion mobility and molecular dynamics. J. Am. Chem. Soc..

[49] Devine PWA, Fisher HC, Calabrese AN, Whelan F, Higazi DR, Potts JR, Lowe DC, Radford SE, Ashcroft AE (2017). Investigating the structural compaction of biomolecules upon transition to the gas-phase using ESI-TWIMS-MS. J. Am. Soc. Mass Spectrom..

[50] Beveridge R, Migas LG, Payne KA, Scrutton NS, Leys D, Barran PE (2016). Mass spectrometry locates local and allosteric conformational changes that occur on cofactor binding. Nat. Commun..

[51] Pacholarz KJ, Burnley RJ, Jowitt TA, Ordsmith V, Pisco JP, Porrini M, Larrouy-Maumus G, Garlish RA, Taylor RJ, de Carvalho LPS, Barran PE (2017). Hybrid mass spectrometry approaches to determine how L-histidine feedback regulates the enzyzme MtATP-phosphoribosyltransferase. Structure.

[52] Dickinson ER, Jurneczko E, Pacholarz KJ, Clarke DJ, Reeves M, Ball KL, Hupp T, Campopiano D, Nikolova PV, Barran PE (2015). Insights into the conformations of three structurally diverse proteins: cytochrome c, p53, and MDM2, provided by variable-temperature ion mobility mass spectrometry. Anal. Chem..

[53] Sinz A, Arlt C, Chorev D, Sharon M (2015). Chemical cross-linking and native mass spectrometry: a fruitful combination for structural biology. Protein Sci..

