# Supplementary material for: DNA Binding and Phosphorylation Regulate the Core Structure of the NF-κB p50 Transcription Factor
Source: J Am Soc Mass Spectrom. 2018 Jun 5;30(1):128–38. doi: 10.1007/s13361-018-1984-0 (PMC6318249; doi:10.1007/s13361-018-1984-0)
Supplement: Supplementary file 1 — (DOCX 1008 kb) [file 13361_2018_1984_MOESM1_ESM.docx]

DNA binding and phosphorylation regulate the core structure of the NF-κB p50 transcription factor

Matthias Vonderach,^a^ Dominic P. Byrne,^b^ Perdita E. Barran,^c^ Patrick A. Eyers,^b^ and Claire E. Eyers^a^

1. Centre for Proteome Research, Department of Biochemistry, Institute of Integrative Biology, University of Liverpool, Crown Street, Liverpool, L69 7ZB, UK
2. Department of Biochemistry, Institute of Integrative Biology, University of Liverpool, Crown Street, Liverpool, L69 7ZB, UK
3. Michael Barber Centre for Collaborative Mass Spectrometry, Manchester Institute of Biotechnology, The University of Manchester, 131 Princess Street, Manchester M1 7DN, UK.

Running title: DNA binding stabilises NF-κB dimers

Address reprint requests to: Professor Claire E. Eyers, Centre for Proteome Research, Department of Biochemistry, Institute of Integrative Biology, University of Liverpool, Crown Street, Liverpool, L69 7ZB, Liverpool, UK.

Email: [CEyers@liverpool.ac.uk](mailto:CEyers@liverpool.ac.uk)

Phone number: +44 151 795 4424


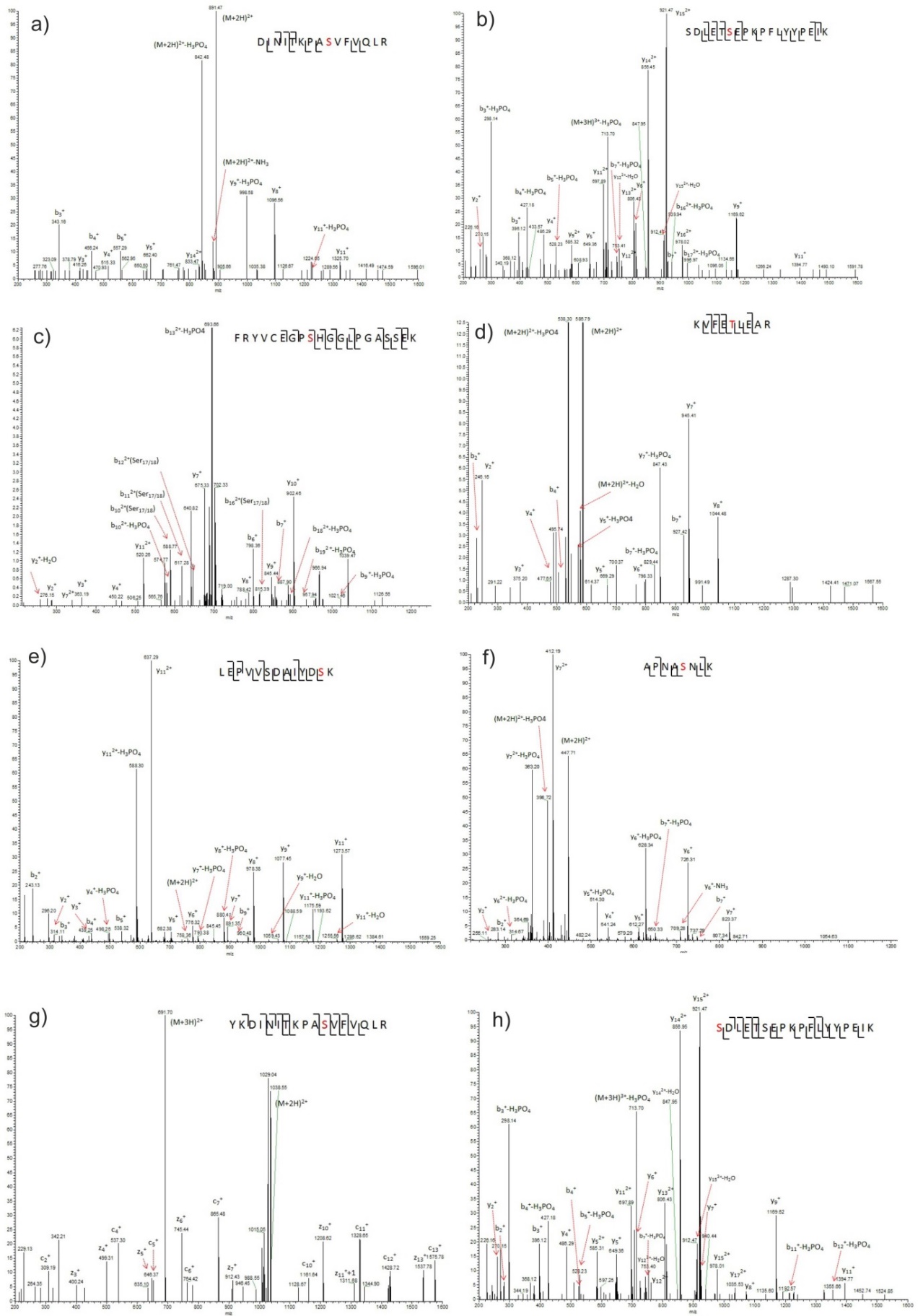


Supplementary Figure 1: Tandem mass spectra of p50 tryptic phosphopeptides. Doubly charged peptide ions derived from the PKAc treated sample: (a) *m/z* 890.97 indicating phosphorylation of Ser328 and (b) *m/z* 746.36 indicating phosphorylation of Ser337. Chk1-treated p50 also yielded phosphorylated tryptic peptide ions at (c) *m/z* 719.98 indicating phosphorylation of Ser65, (d) *m/z* 586.79 representing pThr152, (e) *m/z* 758.35 representing pSer242, (f) *m/z* 447.71 representing pSer248, (g) *m/z* 691.70 representing pSer328, and (h) *m/z* 746.35 representing pSer337. Sites of phosphorylation are in red on the annotated peptide sequence (insets).

Q66IP9|Xenopus_NFKB1 MAQNM-Y-----DIDDAHLETFLRINPLFQEPMYMPSEPHLRNAEGPYIEITEQPKQRGF

Q04861|Chicken_NFKB1 MAGEDPYIMGVSDPQMFAMDQLMGMSTIFNNTGYITSDLPLRTADGPYLQIIEQPKQRGF

P25799|Mouse_NFKB1 MADDDPY--G--TGQMFHLNTAL-THSIFNAELYSP-EIPLST-DGPYLQILEQPKQRGF

F1MKW9|Cow_NFKB1 MAEDDPYLGG--HEQMFHLDP-L-NHTMFSPELFQP-EMPLPTADGPYLQILEQPKQRGF

Q6F3J0|Dog_NFKB1 MAEDDTYLGA--HEQMFHLDP-L-THTIFNPELFQP-EMPLPTADGPYLQILEQPKQRGF

A7XNS1|Pig_NFKB1 MAEDDPYLGG--HDQMFHLDP-L-NHTIFNPELFQP-EMPLPTADGPYLQILEQPKQRGF

F6Z5H5|Marmoset_NFKB1 MAEDDPYLGR--PEQMFHLDPSL-THTIFNPEVFQP-QMALPT-DGPYLQILEQPKQRGF

F7HKH9|Rhesus_macaque MAEDDPYLGR--PEQMFHLDPSL-THTIFNPEVFQP-QMALPT-DGPYLQILEQPKQRGF

H2QPY8|Chimpanzee_NFKB1 MAEDDPYLGR--PEQMFHLDPSL-THTIFNPEVFQP-QMALPTADGPYLQILEQPKQRGF

P19838|Human_p50 **1** MAEDDPYLGR--PEQMFHLDPSL-THTIFNPEVFQP-QMALPT-DGPYLQILEQPKQRGF **55**

** : * : :: : .:*. : . : * . :***::* ********

Q66IP9|Xenopus_NFKB1 RFRYVCEGPSHGGLPGASSEKNRKSYPQIKIHNYVGQVKVVVQLITNSKDIRLHAHSLVG

Q04861|Chicken_NFKB1 RFRYVCEGPSHGGLPGASSEKNKKSYPQVKICNYVGPAKVIVQLVTNGKYVHLHAHSLVG

P25799|Mouse_NFKB1 RFRYVCEGPSHGGLPGASSEKNKKSYPQVKICNYVGPAKVIVQLVTNGKNIHLHAHSLVG

F1MKW9|Cow_NFKB1 RFRYVCEGPSHGGLPGASSEKNKKSYPQVKICNYVGPAKVIVQLVTNGKNIHLHAHSLVG

Q6F3J0|Dog_NFKB1 RFRYVCEGPSHGGLPGASSEKNKKSYPQVKICNYVGPAKVIVQLVTNGKNIHLHAHSLVG

A7XNS1|Pig_NFKB1 RFRYVCEGPSHGGLPGASSEKNKKSYPQVKICNYVGPAKVIVQLVTNGKNIHLHAHSLVG

F6Z5H5|Marmoset_NFKB1 RFRYVCEGPSHGGLPGASSEKNKKSYPQVKICNYVGPAKVIVQLVTNGKNTHLHAHSLVG

F7HKH9|Rhesus_macaque RFRYVCEGPSHGGLPGASSEKNKKSYPQVKICNYVGPAKVIVQLVTNGKNIHLHAHSLVG

H2QPY8|Chimpanzee_NFKB1 RFRYVCEGPSHGGLPGASSEKNKKSYPQVKICNYVGPAKVIVQLVTNGKNIHLHAHSLVG

P19838|Human_p50 **56** RFRYVCEGPSHGGLPGASSEKNKKSYPQVKICNYVGPAKVIVQLVTNGKNIHLHAHSLVG **115**

**********************.*****:** **** .**:***:**.* .********

Q66IP9|Xenopus_NFKB1 KNCEDGICSLTVGPKDTIVGFPNLGILHVTKKKVIEILEARMTDAFKKGHNAALLVHPEL

Q04861|Chicken_NFKB1 KFCEDGVCTVNAGPKDMVVGFANLGILHVTKKKVFETLETRMIDACKKGYNPGLLVHPEL

P25799|Mouse_NFKB1 KHCEDGVCTVTAGPKDMVVGFANLGILHVTKKKVFETLEARMTEACIRGYNPGLLVHSDL

F1MKW9|Cow_NFKB1 KHCEDGVCTVTAGPKDMVVGFANLGILHVTKKKVFETLEARMTDACIRGYNPGLLVHPDL

Q6F3J0|Dog_NFKB1 KHCEDGICTVTAGPKDMVVGFANLGILHVTKKKVFETLEARMTEACTKGYNPGLLVHPDL

A7XNS1|Pig_NFKB1 KHCEDGICTVTAGPKDMVVGFANLGILHVTKKKVFETLEARMTEACIRGYNPGLLVHPDL

F6Z5H5|Marmoset_NFKB1 KHCEDGICTVTAGPKDMVVGFANLGILHVTKKKVFETLEARMTEACIRGYNPGLLVHPDL

F7HKH9|Rhesus_macaque KHCEDGICTVTAGPKDMVVGFANLGILHVTKKKVFETLEARMTEACIRGYNPGLLVHPDL

H2QPY8|Chimpanzee_NFKB1 KHCEDGICTVTAGPKDMVVGFANLGILHVTKKKVFETLEARMTEACIRGYNPGLLVHPDL

P19838|Human_p50 **116** KHCEDGICTVTAGPKDMVVGFANLGILHVTKKKVFETLEARMTEACIRGYNPGLLVHPDL **175**

* ****:*::..**** :***.************:* **:** :* .*:*..****.:*

Q66IP9|Xenopus_NFKB1 NYTNSE---DRPLNEREKEIIRQAATQQSKDIDLSVVRLMFTAFLLDSEGRFTRSLEPVL

Q04861|Chicken_NFKB1 GYLQAEGCGDRQLTEREREIIRQAAVQQTKEMDLSVVRLMFTAFLPDSNGGFTRRLDPVI

P25799|Mouse_NFKB1 AYLQAEGGGDRQLTDREKEIIRQAAVQQTKEMDLSVVRLMFTAFLPDSTGSFTRRLEPVV

F1MKW9|Cow_NFKB1 AYLQAEGGGDRQLTDREKEIIRQAALQQTKEMDLSVVRLMFTAFLPDSTGSFTRRLEPVV

Q6F3J0|Dog_NFKB1 AYLQAEGGGDRQLTDREKEIIRQAALQQTKEMDLSVVRLMFTAFLPDSTGSFTRRLEPVV

A7XNS1|Pig_NFKB1 AYLQAEGGGDRQLTDREKEIIRQAALQQTKEMDLSVVRLMFTAFLPDSTGSFTRRLEPVV

F6Z5H5|Marmoset_NFKB1 AYLQAEGGGDRQLGDREKELIRQAALQQTKEMDLSVVRLMFTAFLPDSTGSFTRRLEPVV

F7HKH9|Rhesus_macaque AYLQAEGGGDRQLGDREKELIRQAALQQTKEMDLSVVRLMFTAFLPDSTGSFTRRLEPVV

H2QPY8|Chimpanzee_NFKB1 AYLQAEGGGDRQLGDREKELIRQAALQQTKEMDLSVVRLMFTAFLPDSTGSFTRRLEPVV

P19838|Human_p50 **176** AYLQAEGGGDRQLGDREKELIRQAALQQTKEMDLSVVRLMFTAFLPDSTGSFTRRLEPVV **235**

* ::* ** * :**.*:***** **:*::************* ** * *** *:**:

Q66IP9|Xenopus_NFKB1 SVPIFDSKAPNASNLKIVRMDRTAGCVTGGEEVYLLCDKVQKDDIQVRFYEEDENGGYWE

Q04861|Chicken_NFKB1 SDAIYDSKAPNASNLKIVRMDRTAGCVTGGEEIYLLCDKVQKDDIQIRFYEEDENGGMWE

P25799|Mouse_NFKB1 SDAIYDSKAPNASNLKIVRMDRTAGCVTGGEEIYLLCDKVQKDDIQIRFYEEEENGGVWE

F1MKW9|Cow_NFKB1 SDAIYDSKAPNASNLKIVRMDRTAGCVTGGEEIYLLCDKVQKDDIQIRFYEEEENGGIWE

Q6F3J0|Dog_NFKB1 SDAIYDSKAPNASNLKIVRMDRTAGCVTGGEEIYLLCDKVQKDDIQIRFYEEEENGGIWE

A7XNS1|Pig_NFKB1 SDAIYDSKAPNASNLKIVRMDRTAGCVTGGEEIYLLCDKVQKDDIQIRFYEEEENGGIWE

F6Z5H5|Marmoset_NFKB1 SDAIYDSKAPNASNLKIVRMDRTAGCVTGGEEIYLLCDKVQKDDIQIRFYEEEENGGVWE

F7HKH9|Rhesus_macaque SDAIYDSKAPNASNLKIVRMDRTAGCVTGGEEIYLLCDKVQKDDIQIRFYEEEENGGVWE

H2QPY8|Chimpanzee_NFKB1 SDAIYDSKAPNASNLKIVRMDRTAGCVTGGEEIYLLCDKVQKDDIQIRFYEEEENGGVWE

P19838|Human_p50 **236** SDAIYDSKAPNASNLKIVRMDRTAGCVTGGEEIYLLCDKVQKDDIQIRFYEEEENGGVWE **295**

* .*:***************************:*************:*****:**** **

Q66IP9|Xenopus_NFKB1 GFGDFSPTDVHRQFAIVFKTPKYKDVNITKAASVFVQLRRKSDYETSEPKPFLYYPEIKD

Q04861|Chicken_NFKB1 GFGDFSPTDVHRQFAIVFKTPKYRDVNITKPASVFVQLRRKSDLETSEPKPFLYYPEIKD

P25799|Mouse_NFKB1 GFGDFSPTDVHRQFAIVFKTPKYKDVNITKPASVFVQLRRKSDLETSEPKPFLYYPEIKD

F1MKW9|Cow_NFKB1 GFGDFSPTDVHRQFAIVFKTPKYKDVNITKPASVFVQLRRKSDLETSEPKPFLYYPEIKD

Q6F3J0|Dog_NFKB1 GFGDFSPTDVHRQFAIVFKTPKYKDVNITKPASVFVQLRRKSDLETSEPKPFLYYPEIKD

A7XNS1|Pig_NFKB1 GFGDFSPTDVHRQFAIVFKTPKYKDVNITKPASVFVQLRRKSDLETSEPKPFLYYPEIKD

F6Z5H5|Marmoset_NFKB1 GFGDFSPTDVHRQFAIVFKTPKYKDVNITKPASVFVQLRRKSDLETSEPKPFLYYPEIKD

F7HKH9|Rhesus_macaque GFGDFSPTDVHRQFAIVFKTPKYKDVNITKPASVFVQLRRKSDLETSEPKPFLYYPEIKD

H2QPY8|Chimpanzee_NFKB1 GFGDFSPTDVHRQFAIVFKTPKYKDINITKPASVFVQLRRKSDLETSEPKPFLYYPEIKD

P19838|Human_p50 **296** GFGDFSPTDVHRQFAIVFKTPKYKDINITKPASVFVQLRRKSDLETSEPKPFLYYPEIKD **355**

***********************.*:****.************ ****************

Q66IP9|Xenopus_NFKB1 KEEVQRKRQKLMPNFSDGYGGSGAGSGGGVGGGLY--GGGGGAGGSAGGGYGFSSFSYNN

Q04861|Chicken_NFKB1 KEEVQRKRQKLMPNFSDGYGG-GS--GAG-GGGMF--GGGGGGAGS---GFSYPSYGYSA

P25799|Mouse_NFKB1 KEEVQRKRQKLMPNFSDSFGG-GSGAGAG-GGGMFGSGGGGGSTGSPGPGYGYSNYGFPP

F1MKW9|Cow_NFKB1 KEEVQRKRQKLMPNFSDSFGG-GSGAGAG-GGGMFGSGGGGGGAGSTGPGYGFPHYGFPT

Q6F3J0|Dog_NFKB1 KEEVQRKRQKLMPNFSDSFGG-GSGAGAG-GGGMFGSGGGGGGAGSTGPGYGFPHYGFPT

A7XNS1|Pig_NFKB1 KEEVQRKRQKLMPNFSDSFGG-GSGAGAG-GGGMFGSGGGGGGAGSTGPGYGFPHYGFPT

F6Z5H5|Marmoset_NFKB1 KEEVQRKRQKLMPNFSDSFGG-GSGAGAG-GGGMFGSGSGGGGTGSTGPGYSFPHYGFPT

F7HKH9|Rhesus_macaque KEEVQRKRQKLMPNFSDSFGG-GSGAGAG-GGGMFGSGGGGGGTGSTGPGYSFPHYGFPT

H2QPY8|Chimpanzee_NFKB1 KEEVQRKRQKLMPNFSDSFGG-GSGAGAG-GGGMFGSGGGGGGTGSTGPGYSFPHYGFPT

P19838|Human_p50 **356** KEEVQRKRQKLMPNFSDSFGG-GSGAGAG-GGGMFGSGGGGGGTGSTGPGYSFPHYGFPT **413**

*****************.:** *: *.* ***:: *.***. ** *:.:. :.:

Q66IP9|Xenopus_NFKB1 YGGLNFHGGPMNSGPCMKHE

Q04861|Chicken_NFKB1 FGGMHFHPGTTKSNAGMKHE

P25799|Mouse_NFKB1 YGGITFHPGVTKSNAGVTHG

F1MKW9|Cow_NFKB1 YGGITFHTGTTKSNAGMKHG

A7XNS1|Pig_NFKB1 YGGITFHAGTTKSNAGMKHG

Q6F3J0|Dog_NFKB1 YGGITFHPGTTKSNAGMKHG

F6Z5H5|Marmoset_NFKB1 YGGISFHPGTTKSNAGMKHG

F7HKH9|Rhesus_macaque YGGITFHPGTTKSNAGMKHG

H2QPY8|Chimpanzee_NFKB1 YGGITFHPGTTKSNAGMKHG

P19838|Human_p50 **414** YGGITFHPGTTKSNAGMKHG **433**

:**: ** * :*.. :.*

**Supplementary Figure 2. Cross-species sequence alignment of p50.**

Thirteen higher eukaryote p50 protein sequences: Human (P19838); Pan troglodytes (Chimpanzee, H2QPY8); Macaca mulatta (Rhesus macaque, F7HKH9); Marmoset F6Z5H5; Pig A7XNS1; Cow, F1MKW9; Dog, Q6F3J0; Mouse, P25799; Chicken, Q04861-3; Xenopus Laevis Q66IP9, were aligned with MUSCLE (3.8). Sites of phosphorylation are highlighted red (serine) or yellow (threonine). Non-conservative substitutions are highlighted cyan.


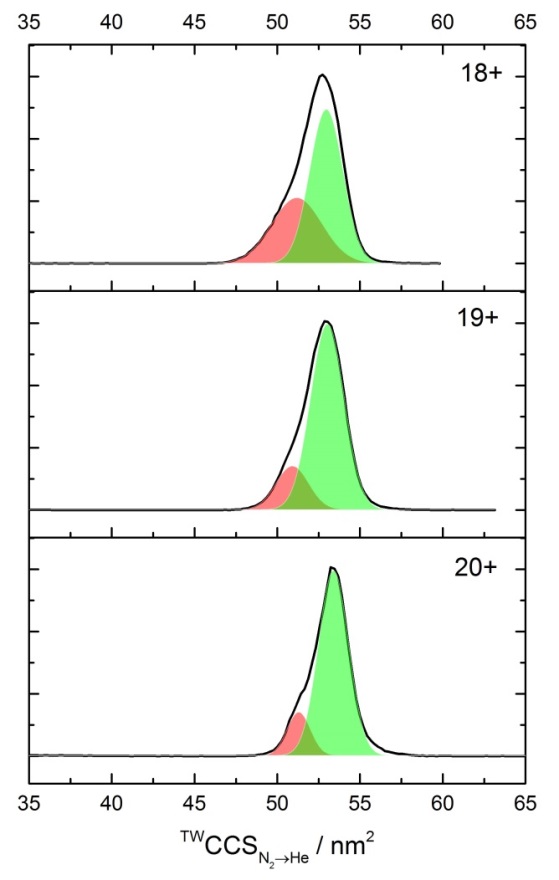

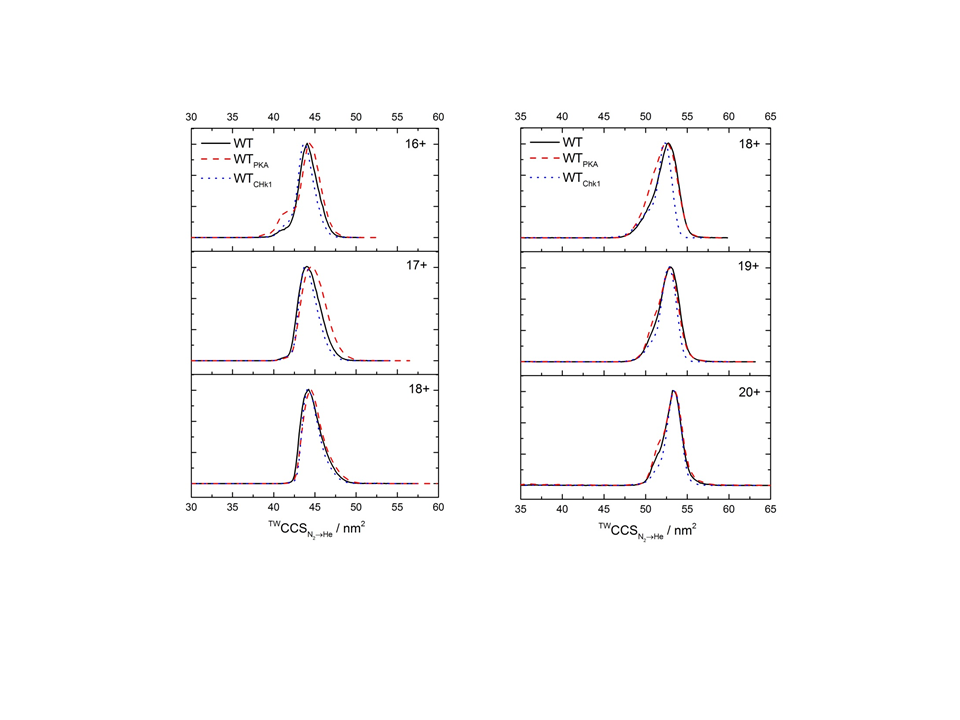


Supplementary Figure 3: CCS distributions of p50 dimer (35-381) before (black line) and after *in vitro* phosphorylation with either PKA_c_ (red) or Chk1 (blue), in the absence (top left) or presence (top right) of κB DNA. (Bottom) Gaussian fitting of the DNA bound dimer indicates two predominant conformers.


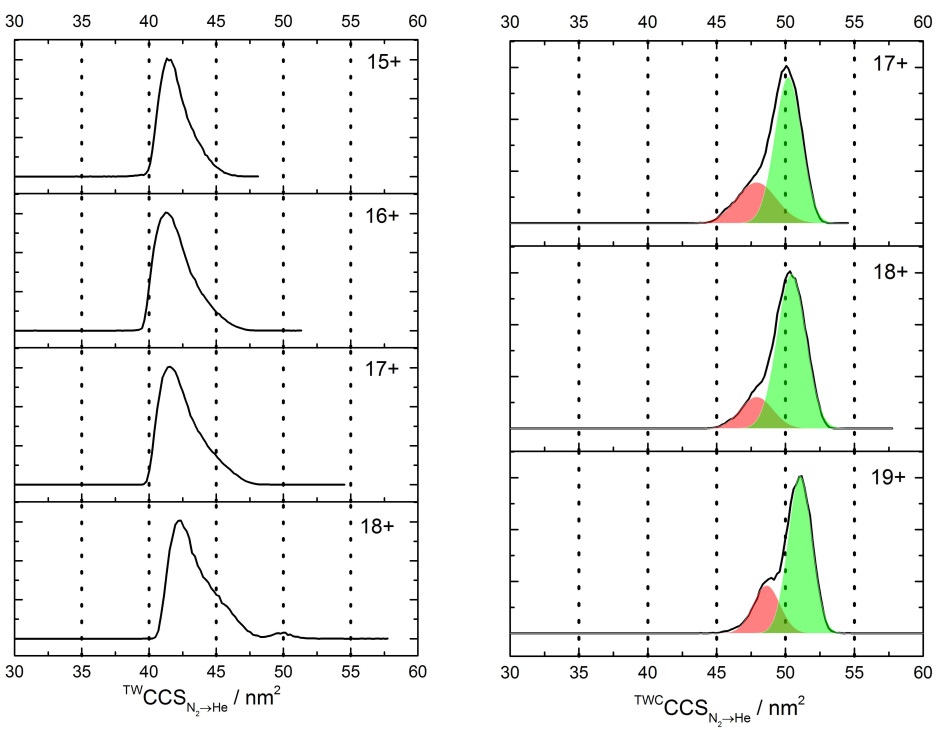
 Supplementary Figure 4: CCS distributions of p50 (39-365) dimer in the absence (left) and presence (right) of κB DNA oligomer. Gaussian fitting of the DNA bound dimer indicates two predominant conformers.

**Supplementary Figure 5: S337D p50 phosphomimetic has decreased preference for dimer formation.** WT or S337D p50 (39-364) were proteolytically separated from the MBP affinity tag using 3C protease, and dimer:monomer ratio evaluated in solution using SEC on a Superdex 200 16/600 column. The position of dimeric p50 (centred at 70 ml column elution volume) and monomeric p50 (centred at 87 ml column elution volume) was confirmed by SDS-PAGE (top). The minor MBP contaminant present in the S337D preparation, which migrates more slowly than p50, is indicated by an asterisk on the gel.


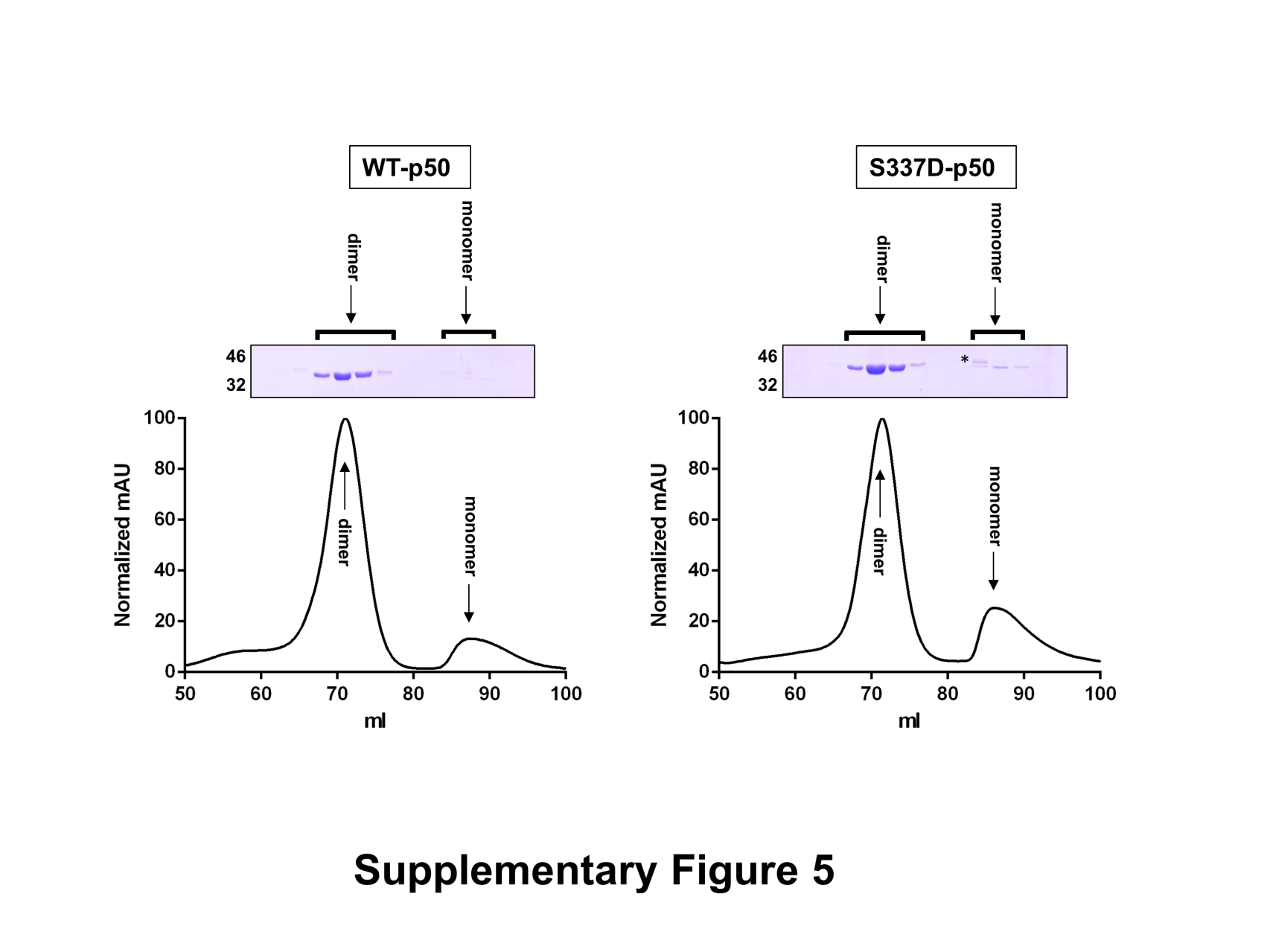


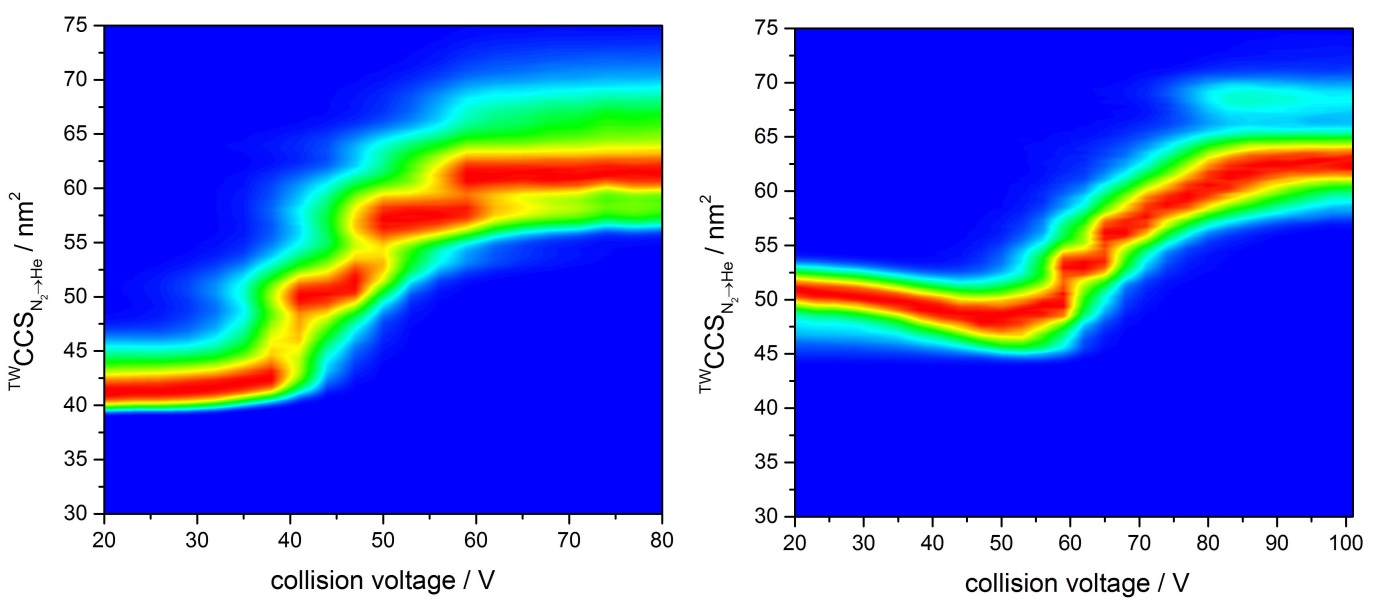


Supplementary Figure 6: DNA binding increases the stability of the p50 dimer. Collision-induced unfolding profiles of the 17+ charge state of WT p50 (39-364) dimer in the absence (left) and presence (right) of DNA indicate markedly different stabilities.
